# BACTERIAL AND FUNGAL INFECTIONS IN PULMONARY TUBERCULOSIS PATIENTS AND THE DRUG SENSITIVITY TEST PATTERNS

**DOI:** 10.21010/Ajidv19i1.3

**Published:** 2024-10-25

**Authors:** SINULINGGA Cita Pujiaty, SINAGA Bintang Yinke Magdalena, SIAGIAN Parluhutan, YUNITA Rina, WAHYUNI Arlinda Sari

**Affiliations:** 1 Department of Pulmonology and Respiratory Medicine, Faculty of Medicine, Universitas Sumatera Utara, Medan, North Sumatra, Indonesia; 2 Department of Pulmonology and Respiratory Medicine, Faculty of Medicine, Universitas Sumatera Utara, Adam Malik Hospital, Medan, North Sumatra, Indonesia; 3 Department of Microbiology, Faculty of Medicine, Universitas Sumatera Utara, Adam Malik Hospital, Medan, North Sumatra, Indonesia; 4 Department of Community Medicine, Faculty of Medicine, Universitas Sumatera Utara, Medan, North Sumatra, Indonesia

**Keywords:** Pulmonary tuberculosis, bacterial growth, fungal growth, mixed infections

## Abstract

**Background:**

Other microbial infections in pulmonary tuberculosis (TB) patients pose significant challenges, complicating treatment outcomes and potentially increasing mortality rates. This study aims to characterize the bacterial and fungal infections profiles in pulmonary TB patients.

**Materials and Methods:**

A retrospective cross-sectional analysis was conducted at Adam Malik Hospital in Medan, Indonesia, from June 2020 to May 2022, involving inpatients diagnosed with drug-sensitive or drug-resistant TB. The data was analyzed from total sampling subjects based on medical records.

**Results:**

From 125 pulmonary TB patients, 64% had drug-sensitive TB (DS-TB) and 36% had drug-resistant TB (DR-TB), with the majority being male and underweight. Microbial analysis showed 33.6% (n=42) of the subjects exhibited bacterial growth, 8.8% (n=11) had fungal growth and 30.4% (n=38) had mixed infection. Of 80 DS-TB patients, 80% had bacterial and fungal infections compared to 60% of 45 DR-TB patients, with *Klebsiella pneumoniae* and *Candida albicans* as the most common microbes. %<Microbial growth patterns were significantly different between DS-TB and DR-TB patients, with 38.8% (n=31) of DS-TB patients displaying mixed bacterial and fungal growth, in contrast to only 15.6% (n=7) of DR-TB patients. There were no significant differences in antibiotic resistance; however, antifungal sensitivity testing revealed a significant difference in response between DS-TB and DR-TB patients, particularly to Flucytosine, Fluconazole, and Micafungin.

**Conclusions:**

There is a considerable presence of bacterial and fungal infections, with *Klebsiella pneumoniae* and *Candida albicans* being the most prevalent. The antifungal sensitivity testing suggesting a need for personalized antifungal treatment strategies between DS-TB and DR-TB patients.

## Introduction

*A*ccording to WHO data from early 2020, tuberculosis remains the leading cause of death from infectious diseases worldwide, even surpassing HIV/AIDS. Tuberculosis was responsible for 1.5 million deaths in 2018 (Attia *et al.*, 2019; Kebede, 2019). In the North Sumatra Province of Indonesia, the estimated number of TB cases in 2021 reached 62,819. The city of Medan alone reported around 1,300 cases (Amirah *et al.*, 2024; Ulfah *et al.*, 2023). The situation is further complicated by the emergence of drug-resistant TB strains, ranging from Multi Drug Resistant Tuberculosis (MDR-TB) to pre-Extensively Drug Resistant Tuberculosis (Pre-XDR-TB) and Extensively Drug Resistant Tuberculosis (XDR-TB) (Susanti *et al.*, 2022).

Bacterial and fungal infections represent significant complications in pulmonary TB patients (Abdulkadir *et al.*, 2020; Sani *et al.*, 2020). A previous study in Ghaemshahr, Iran, from 2007 to 2017 revealed that among 130 pulmonary TB patients, 16 were infected with *Candida albicans* (Amiri *et al.*, 2018). These secondary infections can delay TB healing, lead to further complications, and increase the risk of premature death among pulmonary TB patients (Hosseini *et al.*, 2020). Research by Shimazaki and colleagues in the Philippines indicated that bacterial co-infection was linked to a 1.7-fold increase in early mortality (within two weeks) among pulmonary TB patients (Shimazaki *et al.*, 2018).

The pathogenesis of TB is closely linked to the host’s immune response (Young *et al.*, 2019). Typically, the immune system responds adequately to TB pathogens, limiting bacterial growth and preventing widespread infection. However, much of the tissue damage seen in TB, such as necrosis and lung cavities, is actually a result of the host’s immune response (Kanabalan *et al.*, 2021). Pulmonary TB patients often experience a decline in immune function, attributed to reductions in humoral and cellular immunity (Mori *et al.*, 2021). Lung lesions caused by *Mycobacterium tuberculosis* and associated with prolonged use of anti-tuberculosis drugs, can also target common bacterial pathogens and alter the bacterial landscape along the respiratory tract (Chandra *et al.*, 2022).

Understanding the bacterial profile responsible for secondary pneumonia in active pulmonary TB patients is crucial. It offers essential insights for selecting empirical antibiotic treatments. The growing resistance to commonly used antibiotics presents a significant challenge in choosing appropriate empirical therapies for this patient group. The aim of this study is to characterize the bacterial and fungal infections profiles in patients with pulmonary tuberculosis.

## Materials and Methods

### Study setting, participants, and sampling

We retrospectively reviewed the data of hospitalized patients who underwent GeneXpert MTB/RIF examination and sputum culture simultaneously at Adam Malik Hospital from June 2020 to May 2022. GeneXpert MTB/RIF examination is an integrated molecular diagnostic system that uses real-time polymerase chain reaction (PCR) techniques to detect DNA from *Mycobacterium tuberculosis* complex (MTBC) and genes related to rifampicin drug resistance (Ukwamedua *et al.*, 2019).

The first stage in sputum analysis in a laboratory setting is sputum smear microscopy. A Gram stain is used to distinguish between the two major categories of bacteria: gram-positive and gram-negative. An accurate diagnosis can be made, and an appropriate number of pathogens can be found in the culture by using the Gram stain, which is the initial staining technique used in preliminary bacterial identification. Additionally, the fact that it can particularly address antibiotic medication makes it essential. With the Gram stain, the bacterial species are distinguished into gram-positive and gram-negative groups by the differences in cell walls’ physical and chemical properties. Gram-positive bacteria have extensive peptidoglycan layers on their cell walls that are stained with crystal violet, while some bacteria (gram-negative) have a thinner peptidoglycan coating that is stained red or pink by counterstain. The variables studied in this study were the results of bacterial and fungal sputum cultures and the results of sensitivity tests to antibiotics and antifungals in patients with drug-sensitive pulmonary tuberculosis (PTB) and drug-resistant PTB. Out of 786 data on sensitive and drug-resistant PTB patients who were hospitalized, we excluded 306 due to negative GeneXpert MTB/RIF results, and another 355 due to the absence of culture results.

### Data collection

The variables assessed in this study included both Drug-Sensitive TB (DS-TB) and Drug-Resistant TB (DR-TB), with drug sensitivity testing conducted. From the data collected on TB patients, we extracted information on the occurrence of bacterial and fungal growth or mixed infections in patients who underwent drug sensitivity testing. To assess bacterial growth from the samples, nutrient agar, nutrient broth, and blood agar were employed as culture media. These media were then incubated at 37 degrees Celsius for 24 hours. Gram staining was conducted on the cultured samples to identify bacterial species. For the detection of fungal infections, samples were inoculated onto Sabouraud’s dextrose agar medium. The presence of fungal colonies was confirmed through Lactophenol Cotton Blue staining and direct microscopy. The evaluation of drug sensitivity was carried out using standard microbiological methods.

### Data analysis

The data, compiled from medical records, were entered and analyzed using SPSS for Windows, version 23.0 (IBM, Chicago, USA). The distribution of the samples is presented as n (%) and the associations between groups were assessed using the Chi-square or Fisher’s exact test.

### Ethical approval

This study was approved by Ethical committee of Universitas Sumatera Utara on 25^th^ November 2022 (Approval No: 1175/KEPK/USU/2022).

## Results

In this study, which involved 125 subjects, 64% (n=80) were diagnosed with drug-sensitive tuberculosis (DS-TB), while 36% (n=45) had drug-resistant tuberculosis (DR-TB). Table 1 delineates the demographic and clinical profiles of the participants. The majority of the subjects were male, accounting for 76.8% (n=96), with the largest age groups being those aged 25-34 (28.8%, n=36) and 35-44 (27.2%, n=34). There was a high prevalence of underweight individuals, at 82.4% (n=103). Regarding TB history, a slight majority of the participants, 52.8% (n=66), had no prior history of TB. Comorbidities were present in 25.6% (n=32) of the subjects.

**Table 1:** Sample Characteristics

Variables	Categories	Frequency (n)	Percentage (%)
Sex	Male	96	76.8
	Female	29	23.2
Age	18-24	12	9.6
	25-34	36	28.8
	35-44	34	27.2
	45-54	30	24
	>=55	13	10.4
BMI	Underweight	103	82.4
	Normal	22	17.6
	Overweight	0	0
TB history	Yes	59	47.2
	No	66	52.8
Comorbidity	Yes	32	25.6
	No	93	74.4
TB diagnosis	DS-TB	80	64
	DR-TB	45	36

Table 2 indicates that 27.2% (n=34) of the subjects showed no microbial growth, with a notably higher prevalence observed among DR-TB patients. Bacterial growth was detected in 33.6% (n=42) of the subjects, with DR-TB patients exhibiting a slightly higher incidence. Fungal growth was less prevalent, found in 8.8% (n=11) of the total sample. A significant disparity in mixed microbial growth was noted between DS-TB and DR-TB patients, with 38.8% (n=31) of DS-TB patients displaying mixed growth, in contrast to only 15.6% (n=7) of DR-TB patients. The chi-squared test showed a statistically significant difference (p<0.05) in microbial growth patterns between groups.

**Table 2:** Bacterial and fungal growth based on diagnosis (n=125)

				Total		TB diagnosis				p-value^a^

						DS-TB		DR-TB		

				n	%	n	%	n	%	
Microbial growth	No growth			34	27.2	16	20	18	40	0.018*
	Bacteria			42	33.6	25	31.2	17	37.8	
	Fungal			11	8.8	8	10	3	6.7	
	Mixed			38	30.4	31	38.8	7	15.5	

Total		125	100	80	100	45	100			

a Chi-squared test;

* statistically significant (p<0.05)

Table 3 and Table 4 explore the species of bacterial and fungal growth. The most prevalent Gram-negative bacterium was *Klebsiella pneumoniae*, found in 28.8% (n=36) of the total sample, with a slightly higher incidence in DS-TB patients (30%, n=24) compared to DR-TB patients (26.7%, n=12). Other notable Gram-negative bacteria included *Acinetobacter baumannii* and *Pseudomonas aeruginosa*. The occurrences of Gram-positive bacteria were generally lower, with species such as *Enterococcus casseliflavus* and various *Staphylococcus* and *Streptococcus* species being identified. *Candida albicans* was the most common fungus, present in 20% (n=25) of the total sample, with its prevalence slightly higher among DS-TB patients (21.3%, n=17) than in DR-TB patients (17.8%, n=8). Statistical analyses revealed no significant differences in the prevalence of these bacterial and fungal species between DS-TB and DR-TB patients, indicating a similar pattern regardless of TB drug-resistance status.

**Table 3:** Species of bacterial growth based on diagnosis

		Total (N=125)		TB diagnosis				p-value

				DS-TB		DR-TB		

		n	%	n	%	n	%	
Gram negative bacteria	*Klebsiella pneumoniae*	36	28.8	24	30	12	26.7	0.693^a^
	*Enterobacter cloacae*	4	3.2	3	3.8	1	2.2	1^b^
	*Acinetobacter baumannii*	19	15.2	13	16.3	6	13.3	0.663^a^
	*Pseudomonas aeruginosa*	8	6.4	3	3.8	5	11.1	0.135^b^
	*Escherichia coli ESBL (+)*	6	4.8	4	5	2	4.4	1^b^
	*Aeromonas hydrophila*	1	0.8	1	1.3	0	0	1^b^
	*Stenotrophomonas maltophilia*	2	1.6	2	2.5	0	0	0.535^b^
	*Pandoraea spp*	2	1.6	1	1.3	1	2.2	1^b^
	*Sphingomonas paucimobilis*	1	0.8	1	1.3	0	0	1^b^
Gram positive bacteria	*Enterococcus casseliflavus*	2	1.6	2	2.5	0	0	0.535^b^
	*Granulicatella adiacens*	2	1.6	0	0	2	4.4	0.128^b^
	*Staphylococcus aureus*	1	0.8	1	1.3	0	0	1^b^
	*Staphylococcus haemolyticus*	1	0.8	1	1.3	0	0	1^b^
	*Staphylococcus spp*	2	1.6	0	0	2	4.4	0.128^b^
	*Streptococcus constellatus*	1	0.8	1	1.3	0	0	1^b^
	*Streptococcus mitis*	1	0.8	1	1.3	0	0	1^b^
	*Streptococcus sanguinis*	2	1.6	2	2.5	0	0	0.535^b^
	*Streptococcus parasanguinis*	2	1.6	1	1.3	1	2.2	1^b^
	*Rothia mucilaginosa*	1	0.8	1	1.3	0	0	1^b^
	*Gram (+) Cocci*	1	0.8	1	1.3	0	0	1^b^

a Chi-squared test;

b Fisher exact test

**Table 4:** Species of fungal growth based on diagnosis

		Total (n=125)		TB diagnosis				p-value

				DS-TB		DR-TB		

		n	%	n	%	n	%	
Fungi	*Candida albicans*	25	20	17	21.3	8	17.8	0.641^a^
	*Candida dubliniensis*	3	2.4	2	2.5	1	2.2	1^b^
	*Candida glabrata*	4	3.2	3	3.8	1	2.2	1^b^
	*Candida parapsilosis*	2	1.6	2	2.5	0	0	0.535^b^
	*Candida tropicalis*	9	7.2	8	10	1	2.2	0.155^b^
	*Cryptococcus laurentii*	5	4	5	6.3	0	0	0.159^b^
	*Stephanoascus ciferrii*	4	3.2	3	3.8	1	2.2	1^b^
	*Trichosporon asahii*	1	0.8	1	1.3	0	0	1^b^

a Chi-squared test;

b Fisher exact test

Table 5 presents the antibiotic sensitivity test results for patients diagnosed with DS-TB and DR-TB. The range of antibiotics tested extends from Amikacin to Tigecycline, with the outcomes categorized as sensitive, resistant, or intermediate. For instance, Amikacin exhibited high sensitivity rates in both DS-TB (60.9%, n=39) and DR-TB (70.4%, n=19) patients, with minimal resistance observed. In contrast, antibiotics such as Ampicillin and Cefazolin showed high resistance rates, especially among DS-TB patients (50%, n=32 for Ampicillin; 35.9%, n=23 for Cefazolin). The results underscore the varied responses to antibiotics between groups, with no significant differences in sensitivity or resistance between DS-TB and DR-TB patients detected.

**Table 5:** Sensitivity test results of antibiotics based on diagnosis

	DS-TB (n=64)						DR-TB (n=27)						p value

	*Sensitive*		*Resistant*		*Intermediate*		*Sensitive*		*Resistant*		*Intermediate*		

	n	%	n	%	n	%	n	%	n	%	n	%	
Amikacin	39	60.9	4	6.3	0	0	19	70.4	0	0	0	0	0.482^a^
Ampicillin	1	1.6	32	50	0	0	2	7.4	12	44.4	0	0	0.44^a^
Ampicillin-Sulbactam	21	32.8	21	32.8	3	4.7	9	33.3	7	25.9	2	7.4	0.85^a^
Aztreonam	12	18.8	13	20.3	1	1.6	5	18.5	8	29.6	1	3.7	0.589^a^
Cefazolin	1	1.6	23	35.9	0	0	0	0	14	51.9	0	0	0.36^a^
Cefepime	37	57.8	6	9.4	0	0	15	55.6	4	14.8	0	0	0.745^b^
Ceftazidime	28	43.8	14	21.9	4	6.3	10	37	8	29.6	1	3.7	0.844^a^
Ceftriaxone	6	9.4	31	48.4	7	10.9	3	11.1	10	37	2	7.4	0.64^a^
Ciprofloxacin	18	28.1	29	45.3	0	0	6	22.2	11	40.7	2	7.4	0.224^a^
Cotrimoxazole	28	43.8	18	28.1	0	0	8	29.6	8	29.6	0	0	0.383^b^
Ertapenem	21	32.8	0	0	1	1.6	10	37	1	3.7	0	0	0.426^a^
Gentamicin	31	48.4	18	28.1	0	0	12	44.4	5	18.5	2	7.4	0.165^a^
Meropenem	37	57.8	3	4.7	0	0	17	63	3	11.1	0	0	0.374^a^
Tigecycline	39	60.9	4	6.3	3	4.7	12	44.4	2	7.4	0	0	0.237^a^

a Fisher exact test;

b Chi-squared test

The antifungals tested encompass Amphotericin B, Flucytosine, Fluconazole, Micafungin, and Voriconazole. A significant observation is the consistently high sensitivity to Flucytosine, Fluconazole, and Micafungin in DS-TB patients (59.4%, n=38 for each), contrasted with a sensitivity rate of 33.3% (n=9) in DR-TB patients. This difference, which is statistically significant (p=0.023), indicates a distinct response to these antifungal treatments between groups (Table 6).

**Table 6:** Sensitivity test results of antifungals based on diagnosis

	DS-TB (n=64)						DR-TB (n=27)						p-value

	*Sensitive*		*Resistant*		*Intermediate*		*Sensitive*		*Resistant*		*Intermediate*		

	n	%	n	%	n	%	n	%	n	%	n	%	
Amphotericin B	35	54.7	3	4.7	0	0	9	33.3	0	0	0	0	0.072^a^
Flucytosine	38	59.4	0	0	0	0	9	33.3	0	0	0	0	0.023^b^*
Fluconazole	38	59.4	0	0	0	0	9	33.3	0	0	0	0	0.023^b^*
Micafungin	38	59.4	0	0	0	0	9	33.3	0	0	0	0	0.023^b^*
Voriconazole	34	53.1	4	6.3	0	0	10	37	0	0	0	0	0.095^a^

a Fisher exact test;

b Chi-squared test;

* statistically significant (p<0.05)

## Discussion

Secondary bacterial infection constitutes a significant complication for patients with pulmonary TB (Attia *et al.*, 2019). This study revealed that bacterial and/or fungal infections are common in pulmonary TB, underscoring the importance of incorporating relevant antibiotic/antifungal therapy for respiratory pathogens into TB management strategies. The predominance of male participants (76.8%) and the high incidence of underweight individuals (82.4%) observed in our study are consistent with prior research (Chhabra *et al.*, 2021), which highlights gender disparities in TB prevalence and the link between TB and nutritional status (Dias *et al.*, 2022). Moreover, the age distribution, particularly the concentration of cases in the 25-44 age groups, emphasizes TB’s impact on the economically active segment of the population (Lee *et al.*, 2018; Satyanarayana *et al.*, 2020).

A study conducted by Langbang *et al*. showed that secondary bacterial infection in pulmonary tuberculosis was found in 21 male patients (58.3%) and 15 female patients (40.7%). Out of these, 27 out of 36 cases of secondary bacterial infection came from new cases, amounting to 75% (Langbang *et al.*, 2016). In Nigeria, out of the 141 patients reviewed, there were 79 males and 62 females, with an overall mean age of 35.98±15.93 (Iliyasu *et al.*, 2018).

From the research reported by Liu *et al*. potential bacterial pathogens were more often found in individuals with evidence of malnutrition, as indicated by lower body weight and lower serum albumin levels. Previous evidence suggests that those with poor nutrition, especially those low in protein and zinc, may have impaired cellular immunity (Liu *et al.*, 2022). Data obtained from Dr. Soetomo Hospital, Surabaya, indicated that the average age of fungal co-infection was 44.18 years, with 75 participants being underweight and 73 being of normal weight. Additionally, there were 37 people with comorbid DM and as many as 111 people without comorbid DM (Soedarsono *et al.*, 2020). Meanwhile, a study in India found that TB co-infection with fungal infections in men (15.4%) was higher than in women (14.7%) (Amiri *et al.*, 2018). From the research conducted in Egypt, 8 out of 30 samples were MDR-TB patients with secondary fungal infections, with 4 patients having DM (Osman *et al.*, 2013). In a study conducted by Mathavi and colleagues, it was found that the prevalence of fungal infections was more common in patients who had a low BMI compared to those with a normal or high BMI (Mathavi *et al.*, 2014).

Our findings reveal a notable difference in microbial growth patterns between drug-sensitive and drug-resistant TB patients. Specifically, the higher prevalence of no microbial growth among DR-TB patients could suggest an altered microbial environment or immune response in these individuals, potentially influenced by the complex treatment regimens associated with drug resistance (Young *et al.*, 2019).

The identification of *Klebsiella pneumoniae* as the most prevalent Gram-negative bacterium, particularly in DS-TB patients, raises concerns about hospital-acquired infections and the need for stringent infection control measures (Lin *et al.*, 2021). The presence of various Gram-positive bacteria, although less frequent, highlights the diverse bacterial co-infections to which TB patients are susceptible, further complicating their clinical management (Zaidi *et al.*, 2023).

The slight elevation in *Candida albicans* prevalence among DS-TB patients compared to DR-TB patients might reflect differences in immune status or antibiotic usage patterns between these groups (Chinedum *et al.*, 2018). The absence of significant disparities in the overall prevalence of bacterial and fungal species between DS-TB and DR-TB patients suggests that drug resistance in TB does not necessarily predispose patients to specific bacterial or fungal co-infections (Amiri *et al.*, 2018; Shimazaki *et al.*, 2018).

The Bangalore study revealed that out of 100 TB patients with secondary infections, 20% had secondary infections, 65% had secondary bacterial infections, and 35% had secondary fungal infections (Jose, 2019). Another study conducted in India found that 66.7% of *Candida albicans* isolates were among all fungal isolates growing in pulmonary TB patients (Mathavi *et al.*, 2014). At Patna Medical College Hospital, Bihar, India, from 2011 to 2012, 75 sputum samples were collected from patients suspected of TB. The results showed that *Candida albicans* was isolated in 44.4% of cases, followed by *Aspergillus niger* with a prevalence of 33.3% (Babita and Prabhat, 2016). This finding aligns with research conducted in Surabaya, which found that *Candida albicans* was the most common, accounting for 54.05% (Soedarsono *et al.*, 2020). The elevated susceptibility of MDR-TB patients to fungal infection can be explained by understanding the immunological changes associated with multidrug-resistant TB. It is asserted that the most important host defenses against fungi are neutrophils and alveolar macrophages. IFN-γ produced by T lymphocytes increases the production of nitric oxide and other reactive nitrogen and oxygen radicals from macrophages (Meersseman *et al.*, 2004).

A study by Shimazaki *et al*. in the Philippines showed that bacterial co-infection was associated with a 1.7-fold higher early (two-week) mortality among patients with pulmonary TB. The most likely explanation for this observation is that bacterial co-infection worsens the clinical course of pulmonary TB patients. Their study conducted additional analyses to explore the relationship between pulmonary TB activity and bacterial co-infection. Although higher bacilli count of *M. tuberculosis* were independently associated with increased mortality, no association between bacilli load and the risk of bacterial co-infection was observed. The study data suggest that bacterial co-infection can occur and may worsen the disease. The study also states that bacterial co-infection is a common manifestation of pulmonary TB, and thus antibiotic therapy for respiratory pathogens is part of TB management (Shimazaki *et al.*, 2018).

The antibiotic sensitivity results offer valuable insights into the potential efficacy of various antibiotics against TB-associated bacterial infections. The high sensitivity to Amikacin across both DS-TB and DR-TB patients, contrasted with the pronounced resistance to Ampicillin and Cefazolin, particularly in DS-TB patients, emphasizes the need for personalized antibiotic stewardship in TB treatment ( Nurahmed *et al.*, 2020; Liu *et al.*, 2023). These results contrast with findings from research in hospitals in Guwahati and Shillong, India, which found that Amikacin was the most frequently resistant antibiotic (Langbang *et al.*, 2016), and a study in Nigeria concluded that the most sensitive antibiotics were Levofloxacin, Ceftriaxone, or Chloramphenicol (Iliyasu *et al.*, 2018).

Our analysis did not reveal significant differences in antibiotic sensitivity and resistance patterns between DS-TB and DR-TB patients. This observation may reflect the intrinsic resistance mechanisms of the bacterial species identified, rather than only the TB drug resistance status, warranting further investigation into the interplay between TB treatment regimens and bacterial co-infections (Heidary *et al.*, 2022; Zaidi *et al.*, 2023).

The antifungal sensitivity testing highlighted a significant difference in response to Flucytosine, Fluconazole, and Micafungin between DS-TB and DR-TB patients. This finding indicates a potential influence of TB drug resistance on the susceptibility of fungal co-infections to antifungal treatments, suggesting a crucial interrelationship that requires further exploration (Denning, 2022). The statistically significant difference in sensitivity to certain antifungals between the two patient groups (p=0.023) raises critical questions about the impact of antifungal resistance mechanisms and the role of immune modulation in TB (Pathakumari *et al.*, 2020). These aspects are crucial for developing integrated treatment strategies that address both TB and its associated fungal co-infections (Lee *et al.*, 2021).

## Conclusion

The microbial analysis of DR-TB and DS-TB patients indicated a considerable presence of bacterial and fungal infections, with *Klebsiella pneumoniae* and *Candida albicans* being the most prevalent. The antibiotic sensitivity testing underscored a varied response to antibiotics, although not statistically significant. However, antifungal sensitivity testing revealed a statistically significant difference in the response to Flucytosine, Fluconazole, and Micafungin between DS-TB and DR-TB patients, suggesting the need for personalized antifungal strategies. Given the high incidence of other microbial infections and the variable sensitivity to antibiotics and antifungals, there is a clear need for the implementation of comprehensive diagnostic screenings for other pathogen infections in TB patients at the onset of treatment.

### Conflict of interests

The authors declare that there is no conflict of interest associated with this study.

**List of Abbreviations:**
BMI: Body Mass Index,
DS-TB: Drug Sensitive Tuberculosis,
DR-TB: Drug Resistant Tuberculosis,
DNA: Deoxyribonucleic Acid,
DM: Diabetes Mellitus,
ESBL: Extended Spectrum Beta-Lactamase,
HIV/AIDS: Human Immunodeficiency Virus/Acquired Immunodeficiency Syndrome,
IBM: International Business Machines,
MDR-TB: Multi Drug Resistant Tuberculosis,
MTBC: Mycobacterium tuberculosis complex,
MTB/RIF: Mycobacterium Tuberculosis/Rifampicin,
PCR: Polymerase Chain Reaction,
PTB: Pulmonary Tuberculosis,
SPSS: Statistical Program for Social Science,
spp: species,
TB: Tuberculosis,
USA: United States of America,
WHO: World Health Organization,
XDR-TB: Extensively Drug-Resistant Tuberculosis.
